# Benzyl Isothiocyanate Inhibits Prostate Cancer Development in the Transgenic Adenocarcinoma Mouse Prostate (TRAMP) Model, Which Is Associated with the Induction of Cell Cycle G1 Arrest

**DOI:** 10.3390/ijms17020264

**Published:** 2016-02-22

**Authors:** Han Jin Cho, Do Young Lim, Gyoo Taik Kwon, Ji Hee Kim, Zunnan Huang, Hyerim Song, Yoon Sin Oh, Young-Hee Kang, Ki Won Lee, Zigang Dong, Jung Han Yoon Park

**Affiliations:** 1 Department of Food Science and Nutrition, Hallym University, Chuncheon 200-702, Korea; hanjini@snu.ac.kr (H.J.C.); kgt486@snu.ac.kr (G.T.K.); jihui6657@daum.net (J.H.K.); hyerim0715@hallym.ac.kr (H.S.); yhkang@hallym.ac.kr (Y.-H.K.); 2 WCU Biomodulation Major, Department of Agricultural Biotechnology and Center for Food and Bioconvergence, Seoul National University, Seoul 151-921, Korea; kiwon@snu.ac.kr; 3 The Hormel Institute, University of Minnesota, Austin, MN 55912, USA; ldydo@hanmail.net (D.Y.L.); zgdong@hi.umn.edu (Z.D.); 4 Advanced Institutes of Convergence Technology, Seoul National University, Suwon 443-270, Korea; 5 Key Laboratory for Medical Molecular Diagnostics of Guangdong Province, Dongguan Scientific Research Center, Guangdong Medical University, Dongguan, Guangdong 523808, China; zn_huang@yahoo.com; 6 Department of Molecular Medicine, School of Medicine, Lee Gil Ya Cancer and Diabetes Institute, Gachon University, Incheon 406-799, Korea; with62@gachon.ac.kr; 7 Research Institute of Agriculture and Life Sciences, Seoul National University, Seoul 151-742, Korea

**Keywords:** benzyl isothiocyanate, prostate cancer development, TRAMP, cell cycle arrest

## Abstract

Benzyl isothiocyanate (BITC) is a hydrolysis product of glucotropaeolin, a compound found in cruciferous vegetables, and has been shown to have anti-tumor properties. In the present study, we investigated whether BITC inhibits the development of prostate cancer in the transgenic adenocarcinoma mouse prostate (TRAMP) mice. Five-week old, male TRAMP mice and their nontransgenic littermates were gavage-fed with 0, 5, or 10 mg/kg of BITC every day for 19 weeks. The weight of the genitourinary tract increased markedly in TRAMP mice and this increase was suppressed significantly by BITC feeding. H and E staining of the dorsolateral lobes of the prostate demonstrated that well-differentiated carcinoma (WDC) was a predominant feature in the TRAMP mice. The number of lobes with WDC was reduced by BITC feeding while that of lobes with prostatic intraepithelial neoplasia was increased. BITC feeding reduced the number of cells expressing Ki67 (a proliferation marker), cyclin A, cyclin D1, and cyclin-dependent kinase (CDK)2 in the prostatic tissue. *In vitro* cell culture results revealed that BITC decreased DNA synthesis, as well as CDK2 and CDK4 activity in TRAMP-C2 mouse prostate cancer cells. These results indicate that inhibition of cell cycle progression contributes to the inhibition of prostate cancer development in TRAMP mice treated with BITC.

## 1. Introduction

The American Cancer Society estimated that prostate cancer was the second most important cause of cancer death, surpassed by lung cancer and the most frequently diagnosed cancer in American men [1]. Since prostate cancer occurs mainly in older men, the prevention of prostate cancer during earlier life could be an effective way to reduce the rate of prostate cancer-related deaths. According to Michael B. Sporn [2], cancer chemoprevention is the use of natural or pharmacologic substances to reverse, suppress, or prevent the development of cancer. Recently, much effort has been made to find cancer chemopreventive phytochemicals with plant origins because humans have consumed plant phytochemicals for an extensive period and so they are perceived to be reasonably safe.

Epidemiological evidence indicates that dietary intake of cruciferous vegetables decreases the risk of prostate cancers [3]. In addition, Singh and Singh proposed that cancer prevention with dietary isothiocyanates (ITCs) is ready for clinical translational research [4]. Benzyl isothiocyanate (BITC) is one of the components in cruciferous vegetables with anticancer effects, which have been attributed to an ITC functional group. BITC was reported to exert anti-cancer properties in various cancer cells including breast, pancreatic, gastric, and colon cancer [5,6,7,8]. In prostate cancer cells, BITC was reported to induce apoptosis associated with Bcl-xL phosphorylation [9]. Recently, BITC was also reported to induce protective autophagy in human prostate cancer cells via inhibition of the mTOR signaling pathway [10].

In addition to reduced induction of apoptosis, cell cycle deregulation has been acknowledged as a hallmark of cancer progression in most malignant tumors [11]. Several compounds from cruciferous vegetables which have anti-cancer properties have been shown to inhibit cell cycle progression and induce apoptosis *in vitro* and *in vivo* [12,13,14]. Cyclin-dependent protein kinases (CDKs) are the major regulators of the cell cycle [15], and bind to various regulatory subunits known as cyclins. Cyclins are closely associated with cell cycle progression, and provide domains essential for enzymatic activity (reviewed in [16]). The activities of cyclin-CDK complexes are controlled by CDK inhibitors (CKIs). These CKIs, such as p21^CIP1/WAF1^ and p27^KIP1^ bind to cyclin-CDK complexes, rendering them inactive (reviewed in [17]). These comprehensive regulatory mechanisms prevent cell cycle progression when DNA damage or other conditions could cause harm to the cell. Therefore, defining the dietary compounds that have functional roles in the regulation of cell cycle progression during cancer development would be a good strategy for cancer prevention research.

Transgenic adenocarcinoma of the mouse prostate (TRAMP) model has been used to study the effects of phytochemicals on prostate cancer development and progression [12,13,18]. In these mice, carcinogenesis occurs site specifically in the prostate due to the expression of a simian virus 40 large tumor antigen (SV40 Tag)-coding region directed by the prostate-specific rat probasin promoter [19]. In the present study, we examined whether BITC inhibits prostate cancer development in TRAMP mice. We demonstrate, for the first time, that oral administration of BITC attenuates prostate cancer development in an autochthonous mouse tumor model. Our results indicate that the inhibition of cell cycle progression may be an important mechanism by which BITC inhibits prostate cancer development in TRAMP mice.

## 2. Results

### 2.1. BITC Inhibits Prostate Cancer Development in TRAMP Mice

**Figure 1:**
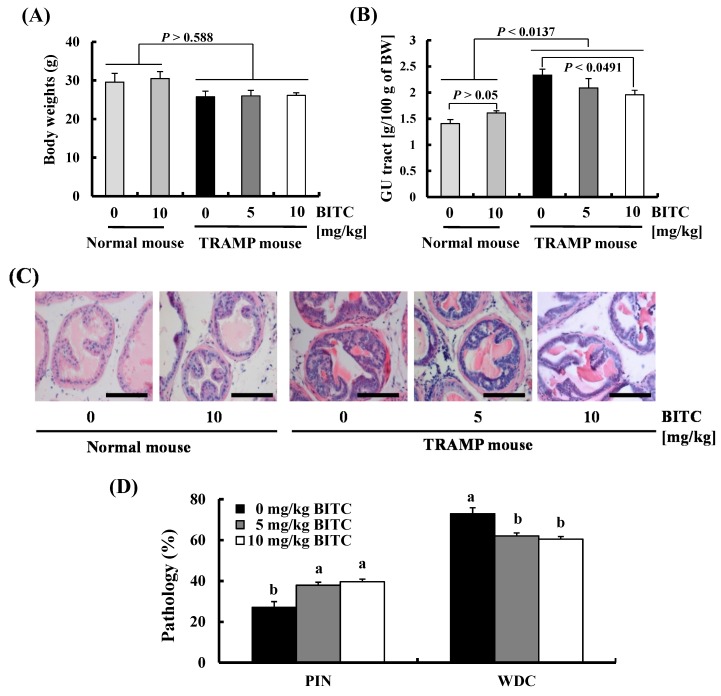
BITC administration delays prostate cancer development in TRAMP mice. Male TRAMP mice and their nontransgenic littermates at five weeks of age were randomly divided into control and BITC-treatment groups and gavage-fed with 0 (vehicle), 5, or 10 mg/kg of BITC every day. At 24 weeks of age, all mice were sacrificed, the GU tracts were excised from the mice and weighed. (**A**) Body weights and (**B**) the GU tract weights; (**C**) Representative photographs of the H and E stained DP from each group (×200); (**D**) The incidence of the prostatic intraepithelial neoplasia and adenocarcinoma of the prostate in TRAMP mice. Data represents the mean ± SEM (*n* = 5). Means without a common letter differ, *p* < 0.05. GU tract, genitourinary tract; PIN, prostatic intraepithelial neoplasia; WDC, well-differentiated carcinoma. Scale bar, 100 μm.

In order to examine whether BITC administration suppresses prostate cancer development, we gavage-fed 5-week old TRAMP mice and their non-transgenic (normal) littermates with BITC for 19 weeks. BITC administration (at 5 or 10 mg/kg body weight) did not affect body weights in either the TRAMP mice or normal mice (Figure 1A). At the time of sacrifice (at 24 weeks of age), there was no considerable difference in organ (liver, lung, and spleen) weights between these groups (Table 1). Additionally, the levels of creatinine and activities of aspartate aminotransferase (AST) and alanine aminotransferase (ALT) in the sera were not increased by BITC administration (Table 2). These results indicate that the chronic administration of BITC (5 or 10 mg/kg/day) was not toxic to the kidney or liver in mice. It has been reported that the genitourinary (GU) tract containing the bladder, urethra, seminal vesicles, ampullary gland, and prostate becomes enlarged as a function of cancer progression in TRAMP mice [20]. The weights of the GU tract were higher in TRAMP mice as compared to non-transgenic mice and this increase was suppressed by BITC feeding (Figure 1B). Sections of the GU tract were stained with hematoxylin and eosin (H and E) to examine the effects of BITC on the pathologic progression of autochthonous prostate cancer in the TRAMP model. At 24 weeks of age, well-differentiated carcinoma (WDC) was a predominant feature in the dorsolateral lobes of the prostate (DP) in vehicle-fed TRAMP mice. In TRAMP mice administered 5 and 10 mg/kg BITC, the number of lobes with prostatic intraepithelial neoplasia (PIN) were higher and those with WDC were lower as compared to those in vehicle-fed TRAMP mice (Figure 1C,D). These results indicate that BITC administration delays prostate cancer development.

### 2.2. BITC Inhibits Cell Cycle Progression in the DP in TRAMP Mice

**Figure 2:**
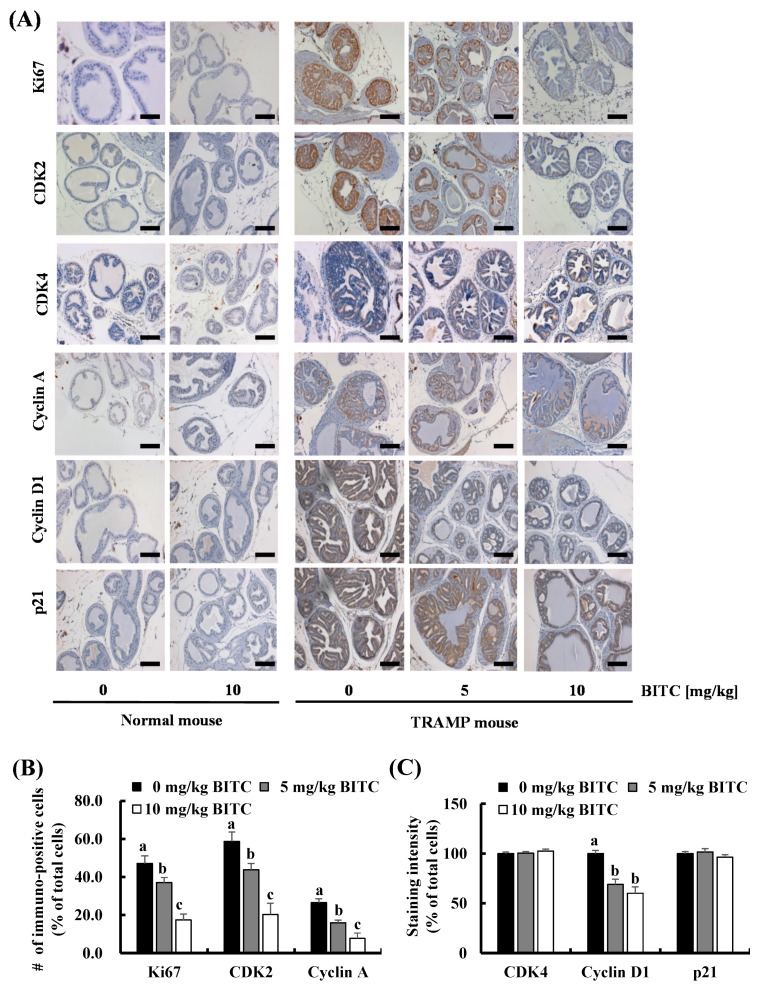
BITC administration reduces the expression of Ki67, CDK2, cyclin A, and cyclin D1 in the prostate epithelium. The prostate sections were immunohistochemically stained using a Ki67, CDK2, CDK4, cyclin A, cyclin D1, or p21 antibody. (**A**) Representative photographs of DAB-stained tissue specimens; (**B**) The number of immune-positive cells were counted and expressed as a percentage of total cells; (**C**) The staining intensity was quantified and the control groups (0 mg/kg BITC-fed TRAMP mice) were set as 100%. Data represents the mean ± SEM (*n* = 5). Means without a common letter differ, *p* < 0.05. Scale bar, 100 μm.

Immunohistochemical staining of the DP revealed that the number of Ki67^+^ cells (proliferating cells) was markedly increased in the DP of TRAMP mice, which was suppressed by BITC administration (Figure 2). Therefore, we next examined whether BITC alters the expression of proteins involved in the regulation of cell cycle progression. The expression of CDK2, CDK4, cyclin A, and cyclin D1, as well as p21 was induced in the DP of TRAMP mice. The expression of these proteins in normal mice was negligible under the conditions of this experiment. The number of CDK2^+^, cyclin A^+^, and cyclin D1^+^ cells in the DP of TRAMP mice was significantly decreased by BITC administration. However, the expression of CDK4 and p21 in the DP of TRAMP mice was not changed by BITC feeding (Figure 2). We could not detect TUNEL-positive apoptotic cells in the DP of either normal or TRAMP mice (data not shown). These results indicate that the BITC-mediated inhibition of prostate carcinogenesis is due, at least in part, to the suppression of cell cycle progression.

### 2.3. BITC Induces G1 Cell Cycle Arrest in TRAMP-C2 Cells

Previous *in vitro* cell culture work has shown that BITC exerts anticancer effects by inducing apoptosis and G2/M cell cycle arrest in various cancer cells including breast cancer, lung cancer, pancreatic cancer and leukemia cells at concentrations between 2 to 100 µmol/L [21,22,23,24,25]. In previous studies involving human prostate cancer cells, BITC has been reported to induce apoptosis and DNA damage in DU145 cells [26].

**Figure 3:**
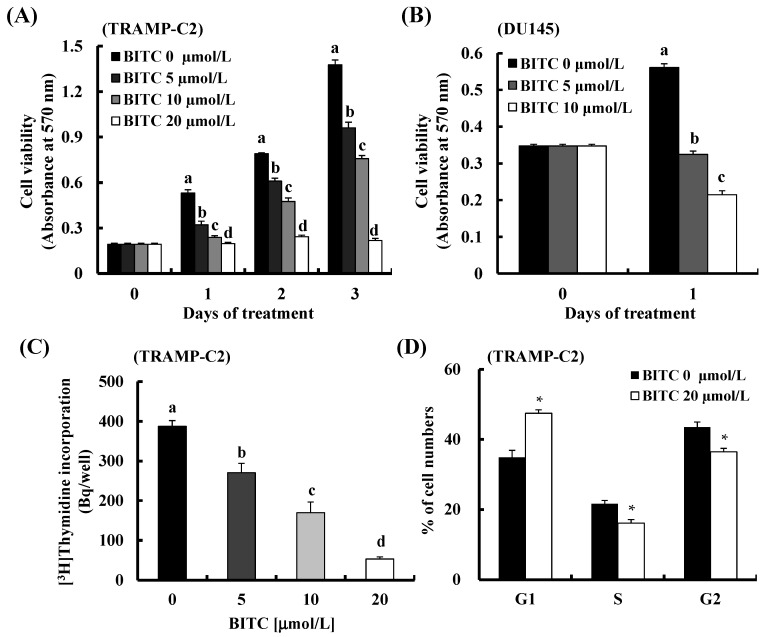
BITC inhibits cell proliferation and induces G1 cell cycle arrest in TRAMP-C2 cells. TRAMP-C2 or DU145 cells were plated in 24-well plates at 5 × 10^4^ cells/well. Twenty-four hours after plating, the monolayers were serum-deprived in DMEM/F12 containing 1% charcoal-stripped FBS for 6 h. (**A**) TRAMP-C2 cells were treated with 0, 5, 10, or 20 µmol/L BITC for one, two, and three days; (**B**) DU145 human prostate cancer cells were treated with 0, 5, or 10 µmol/L BITC for one day. (**A**,**B**) Viable cell numbers were estimated by MTT assay. Each bar represents the mean ± SEM (*n* = 6); (**C**) TRAMP-C2 cells were treated with 0, 5, 10, or 20 µmol/L BITC in the presence of [^3^H]thymidine for 3 h. Each bar represents the mean ± SEM (*n* = 6); (**D**) TRAMP-C2 cells were incubated for 3 h in serum-deprivation medium containing BITC (0 or 20 µmol/L). The nuclei were stained with propidium iodide and the cell cycle was analyzed via flow cytometry. Each bar represents the mean ± SEM (*n* = 5). (**A**–**C**) Means without a common letter differ, *p* < 0.05; (**D**) * *p* < 0.05 as compared with the control group.

As our *in vivo* data showed that BITC feeding inhibited prostate cancer development and also decreased the expression of cell cycle-related proteins in TRAMP mice, we next determined whether BITC directly inhibits prostate cancer cell proliferation in TRAMP-C2 cells (established from a prostate tumor of a TRAMP mouse) [27]. Results of a 3-(4,5-dimethylthiazol-2-yl)-2,5-diphenyltetrazolium bromide (MTT) assay showed that BITC (5–20 µmol/L) reduced the number of viable TRAMP-C2 prostate cancer cells in a dose-dependent manner (Figure 3A). We also observed that BITC treatment reduced the number of viable DU145 human prostate cancer cells (Figure 3B), which is consistent with a previous report [26]. [^3^H]Thymidine incorporation assay results revealed that BITC markedly and dose-dependently inhibited DNA synthesis of TRAMP-C2 cells within 3 h (Figure 3C). In order to determine whether BITC regulates cell cycle progression, the cells were cultured with 0 or 20 µmol/L BITC for 3 h and stained with propidium iodide. An increase in the percentage of cells in the G1 phase was detected after BITC treatment and the G1 phase accumulation was accompanied by a corresponding reduction in the percentage of cells in the S and G2/M phases (Figure 3D). Taken together, these results indicate that the inhibition of cell cycle progression plays a role in BITC inhibition of prostate cancer development.

### 2.4. BITC Inhibits the Expression of Cyclins and the Activity of CDKs in TRAMP-C2 Cells

**Figure 4:**
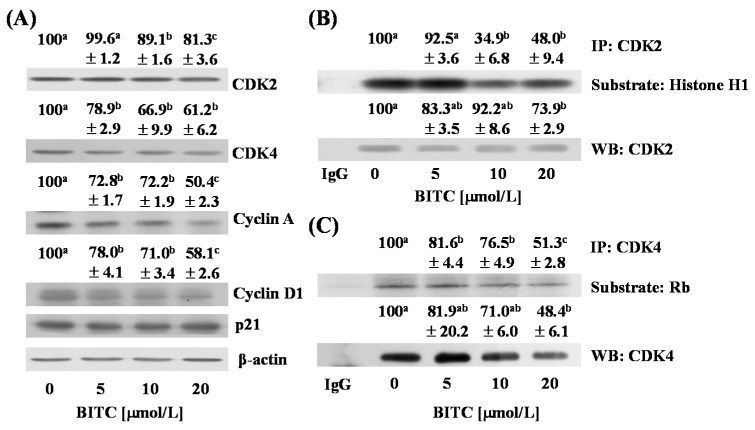
BITC reduces the expression of cyclin A and cyclin D1 and inhibits the activity of CDK2 and CDK4 in TRAMP-C2 mouse prostate cancer cells. Cells were plated in 100 mm dishes at 1 × 10^6^ cells/dish. Twenty-four h after plating, the monolayers were serum-deprived in serum-deprivation medium for 6 h. Cells were then incubated for 3 h with serum-deprivation medium containing various concentrations of BITC (0, 5, 10, or 20 µmol/L); (**A**) The expression of CDK2, CDK4, cyclin A, and cyclin D1 were estimated by immunoblotting. The relative intensity of each band (normalized with its own β-actin) is shown above each band; (**B**,**C**) Cell lysates were incubated with an anti-CDK2 (**B**) or an anti-CDK4 (**C**) antibody and the immune complexes were precipitated with protein A sepharose. For the *in vitro* kinase assay, immunoprecipitated proteins were incubated with a substrate (**B**: Histone H1, **C**: retinoblastoma protein (Rb)) and [γ-^32^P]ATP. Each sample was subjected to SDS-PAGE and the gel was dried. The bands were visualized by autoradiography. For Western blot analysis, immunoprecipitated proteins were analyzed by Western blotting with an anti-CDK2 (**B**) or an anti-CDK4 (**C**) antibody. The relative intensity of each band was quantified and the control (0 μmol/L BITC) levels were set at 100%. Data denotes the mean ± SEM (*n* = 3). Means without a common letter differ, *p* < 0.05.

As BITC reduced DNA synthesis and induced G1 phase arrest (Figure 3C,D), we next examined whether BITC directly regulates the expression of proteins involved in the regulation of G1 cell cycle progression of TRAMP-C2 cells. Consistent with the *in vivo* results, treatment of cells with BITC resulted in a significant reduction in the levels of CDK2, cyclin A, and cyclin D1, whereas the levels of p21 were not altered in BITC-treated cells (Figure 4A). The levels of CDK4 were decreased by BITC treatment in TRAMP-C2 cells, which was inconsistent with the *in vivo* results in which the expression of CDK4 was not altered in the DP of BITC-treated mice (Figure 2). *In vitro* kinase assay results revealed that the activity of CDK2 and CDK4 was reduced by BITC treatment, indicating that the reduced levels of CDKs and cyclins contributed to decreases in CDK activity (Figure 4B,C).

## 3. Discussion

Since epidemiological evidence indicates that dietary intake of cruciferous vegetables decreases the risk of various cancers including prostate cancers [3,28], chemoprevention by dietary ITCs derived from cruciferous vegetables is regarded as an efficient strategy for cancer prevention. Among various ITCs, phenethyl ITC [29] and sulforaphane [30] are known to possess chemopreventive properties against prostate cancer. *In vitro* studies indicate that BITC also exerts anticancer properties in various cancer cells including prostate cancer cells. Previous *in vivo* studies have reported that BITC administration inhibits xenograft tumor growth of leukemia, breast cancer, and melanoma cells in mice [25,31,32,33]. However, the antitumor activity of BITC on the mouse prostate tumor model has not been studied yet. Compared with xenograft models, the TRAMP model is a very high-penetrance model and is difficult to control. However, the TRAMP model is autochthonous, is immune-competent, and shows a pattern of prostate cancer development and progression similar to that seen in the clinical disease [34]. Therefore, we used TRAMP mice to investigate the inhibitory effects of BITC on the development and progression of prostate cancer. In the present study, we showed that oral administration of BITC inhibits prostate cancer development (Figure 1C,D) and decreases cell proliferation indices (Figure 2) in the DP in TRAMP mice. Our results indicate that BITC administration (at 5 or 10 mg/kg body weight) neither affects body weight (Figure 1A) nor shows evidence of kidney or liver injury in mice (Table 1 and Table 2). The present results clearly indicate that the administration of BITC (5–10 mg/kg body weight) delays prostate cancer development without causing a noticeable side effect in TRAMP mice. Similar to our results, Warin *et al.* [35] reported that long-term (25 weeks) administration of BITC (at 1 or 3 mmol/kg diet) inhibited mammary hyperplasia incidence and burden in MMTV-neu mice without causing side effects. The present results suggest that BITC can be developed as a cancer chemopreventive agent for prostate cancer.

In this study, we did not examine the effect of BITC on the survival rate of TRAMP mice. Recently, it was reported that phenethyl ITC, which is a structural analog of BITC, increased the survival rate of mice bearing metastatic breast tumors in the brain [36], suggesting that BITC inhibition of tumor development and progression may increase the survival rate of TRAMP mice. Further studies are needed to determine whether BITC increases the survival rate in various tumor models, including the TRAMP model.

Although there are no research results reporting the antitumor activity of BITC on an animal prostate tumor model, BITC administration has been reported to decrease the number of Ki67^+^ cells and to increase that of TUNEL^+^ apoptotic cells in several other tumor xenograft models [25,31] and in the MMTV-neu transgenic model [35]. We also observed that BITC administration decreases the number of Ki67^+^, CDK2^+^, cyclin A^+^, and cyclin D1^+^ cells in the DP of TRAMP mice (Figure 2). Furthermore, *in vitro* cell culture results revealed that BITC treatment inhibited DNA synthesis (Figure 3C), and decreased the expression of CDK2, CDK4, cyclin A, and cyclin D1, as well as the activity of CDK2 and CDK4 in TRAMP-C2 cells (Figure 4). Several studies showed that BITC administration induces apoptosis in tumor tissues of mouse tumor models [25,31,35]. However, contrary to our expectations, we could not detect changes in apoptosis in the DP of TRAMP mice administered with BITC. Under Hoechst staining to detect apoptosis in the cell culture, BITC did not induce nuclear condensation and fragmentation at the concentrations used in the present experiment (0–20 μmol/L) (data not shown). TRAMP mice have been reported to exhibit a high level of nuclear TRP53 [19], but Western blot results revealed that BITC treatment did not induce the expression of p21 (Figure 4A) or Bax (data not shown), downstream targets of p53. Olvera-Caltzontzin *et al.* [37] reported that iodine increased p53 mRNA expression but had no effect on p21 in tumors from TRAMP mice. TRAMP mice develop autochthonous prostatic tumors that express SV40 Tag. Since SV40 Tag inactivates p53 [38], and p53 plays an important role in cell cycle progression and apoptosis [39], TRAMP mice and TRAMP-C2 cells may not be good models to study the expression of p53 and p53 downstream target proteins.

*In vitro* cell culture results revealed that BITC treatment inhibited the activities of CDK2 and CDK4 (Figure 4B,C). As BITC treatments also reduced the levels of cyclins and CDKs, and the degrees of decreases in CDK activities and in the levels of cyclins and CDKs are similar (Figure 4), it is reasonable to conclude that decreases in the levels of CDKs and cyclins are mainly responsible for the decreases in CDK activities. On can ask whether BITC directly inhibits CDK activity, because several phytochemicals, including ITC, can bind to cellular proteins and BITC can bind to CDKs. However, the computational calculations from both shape screening and molecular modeling indicated that CDKs are unlikely to be the direct target of BITC. The results of shape screening using the Phase module (version 4.0, Schrödinger, LLC, New York, NY, USA, 2014) revealed that BITC had low overall shape similarities with over one hundred ligands co-localized with CDKs from the Protein Data Bank (PDB). Indeed, the highest similarity score was 0.534 for the ligand LZ1 (PDB id 2VTA [40]). In addition, protein-ligand docking simulations using Glide (version 6.4, Schrödinger, LLC, New York, NY, USA, 2014) showed that the binding affinities for BITC and CDKs were low, ~6 kcal/mol, markedly lower than those between the real binders and CDKs (unpublished observation by Huang Z and Dong Z). From these results it is reasonable to conclude that BITC inhibits CDK activities by inhibiting CDK and cyclin expression rather than directly inhibiting their activities.

**Figure 5:**
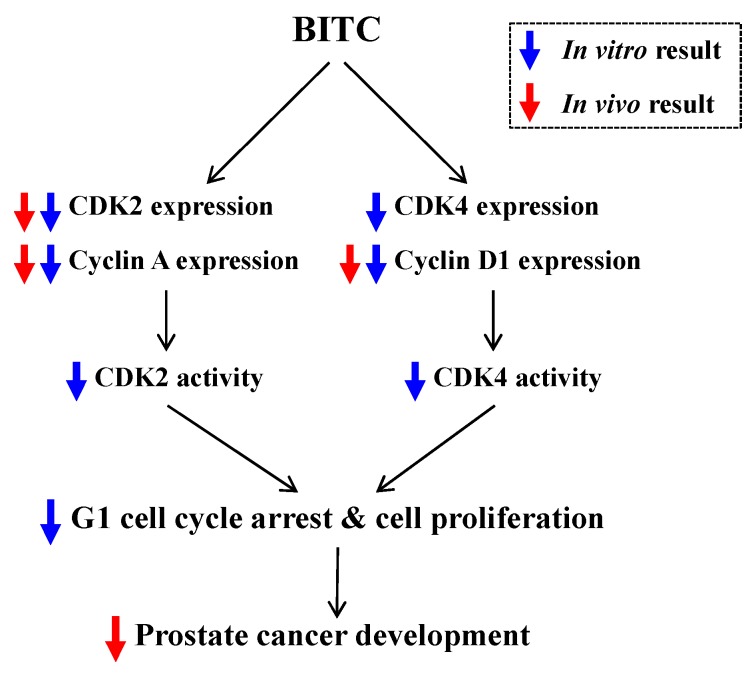
Proposed mechanisms by which BITC inhibits prostate cancer development. *In vitro* cell culture results (blue arrow) revealed that BITC treatment decreases the expression of CDK2, CDK4, cyclin A, and cyclin D1, as well as the activity of CDK2 and CDK4, thereby resulting in the induction of G1 cell cycle arrest. *In vivo* results (red arrow) revealed that BITC administration decreases the expression of CDK2, cyclin A, cyclin D1, and the number of Ki67 (a proliferation marker)-positive cells in the prostatic tissue, resulting in decreases in prostate cancer development.

In conclusion, oral administration of BITC delays prostate cancer development in TRAMP mice. Cell proliferation, as well as the expression of CDK2, cyclin A, and cyclin D1, was markedly reduced in prostate tissues of BITC-fed TRAMP mice. *In vitro* cell culture results revealed that BITC directly induces a reduction in the expression of CDKs and cyclins and thereby reduces CDK’s activity, leading to the induction of G1 cell cycle arrest in TRAMP-C2 cells (Figure 5). These new findings using *in vivo* and *in vitro* prostate cancer models indicate that BITC may be a potent chemopreventive agent for prostate cancer and warrant future studies including human studies. Moreover, further studies are needed to determine the bioavailability and tissue distribution of BITC *in vivo*.

## 4. Materials and Methods

### 4.1. Materials

The following reagents and chemicals were purchased from the indicated suppliers: anti-β-actin antibody and BITC from Sigma (St. Louis, MO, USA); horseradish peroxidase-conjugated anti-rabbit and anti-mouse IgG from Amersham (Arlington Heights, IL, USA); creatinine assay kit from Bio Vision (Milpitas, CA, USA); ALT and AST assay kits from Thermo Fisher Scientific Inc. (Middletown, VA, USA). Antibodies (anti-p21^WAF1/CIP1^, anti-CDK2, anti-CDK4, anti-cyclin A, and anti-cyclin D1) were ordered from Santa Cruz Biotechnology (Santa Cruz, CA, USA).

### 4.2. Animals and Treatments

All animal experiments were approved by the Animal Care and Use Committee of Hallym University (approval number: Hallym2011-08, Chuncheon, Korea) and performed according to the University’s Guidelines for the Care and Use of Laboratory Animals. Heterozygous TRAMP female mice (purchased from the Jackson Laboratory, Bar Harbor, ME, USA) were bred to nontransgenic C57BL/6 normal male mice (The Jackson Laboratory). A rodent chow (Superfeed Co., Wonju, Korea) and water were given *ad libitum* [12]. Newborn mice were weaned and genotyped at four weeks of age as recommended by the Mouse Breeding Strategies Manual of Jackson Laboratory. Mouse tail DNA was isolated with an Extract-N-Amp™ Tissue PCR kit (Sigma) and the TRAMP animals were genotyped via PCR-based DNA screening, as described previously [41]. Male TRAMP mice and their nontransgenic littermates at five weeks of age were randomly divided into control and BITC-treatment groups and gavage-fed with 0 (vehicle), 5, or 10 mg/kg of BITC every day (TRAMP, 5 mice/group; normal, 6 mice/group). BITC was dissolved in dimethyl sulfoxide (DMSO) and diluted with corn oil at a ratio of 1:99 (*v*/*v*). All mice were sacrificed at 24 weeks of age, and the GU tract was isolated, weighed, and then fixed in 4% paraformaldehyde. The liver, lung and spleen were collected and weighed. Blood was collected and sera were assayed to determine the levels of creatinine and activities of AST or ALT according to the manufacturers’ instructions.

### 4.3. Immunohistochemical Analysis

The prostate tissues were embedded in paraffin, sectioned at 5 µm, and stained with H and E for routine histopathologic evaluation [42]. Immunohistochemical assays were conducted as described previously [12]. Briefly, endogenous peroxidases were blocked in 3% hydrogen peroxide. The sections were blocked with PBS containing 5% BSA and incubated with their relevant antibodies at 1:200 dilutions overnight at 4 °C. The sections were then stained using a DAKO LSAB+ System-HRP Kit (DAKO Corporation, Carpinteria, CA, USA) and counterstained with Harris hematoxylin. Apoptotic cells were identified via terminal dUTP nick-end labeling (TUNEL) staining using a DeadEnd^TM^ Fluorometric TUNEL System (Promega, Madison, WI, USA).

### 4.4. Cell Culture

The TRAMP-C2 and DU145 cells (ordered from the American Type Culture Collection, Manassas, VA) were grown in monolayer cultures in Dulbecco’s Modified Eagle’s Medium/Nutrient Mixture F-12 (DMEM/F12) containing 100 mL/L fetal bovine serum (FBS). The culture medium was supplemented with 100,000 U/L of penicillin and 100 mg/L of streptomycin (Biowhittaker, Walkersville, MD, USA). To determine the effects of BITC, we plated TRAMP-C2 or DU145 cells in multi-well plates. After 24 h, cells were serum-deprived for 6 h in DMEM/F12 supplemented with 1% charcoal-stripped FBS. The cells were then treated with various concentrations of BITC. BITC was dissolved in DMSO, and all cells were treated with DMSO to a final concentration of 0.01%. Cell viability was assessed by MTT assay.

### 4.5. (^3^H)Thymidine Incorporation

Thymidine incorporation assay was performed as previously described [43]. Briefly, TRAMP-C2 cells were plated in 96-well plates at 5000 cells/well, serum-deprived, and treated with various concentrations of BITC. [^3^H]Thymidine (0.5 µCi/well) was simultaneously added, and the cells were incubated for 3 h to measure incorporation into the DNA.

### 4.6. Flow Cytometry Analysis of Cell Cycle Distribution

TRAMP-C2 cells were plated in 100 mm dishes at a concentration of 1 × 10^6^ cells/dish in DMEM/F12 containing 10% FBS. Twenty-four h after plating, the cells were serum-deprived for 6 h and treated with 0 or 20 µmol/L BITC for 3 h. The cells were fixed in ethanol and treated with 0.5 g/L RNase. The nuclei were then stained with 0.5 g/L propidium iodide and subjected to fluorescence-activated cell sorting analysis (FACS) using FACScan (Becton Dickinson, Franklin Lakes, NJ, USA). The data were analyzed using Modfit version 1.2 software (Becton Dickinson, Franklin Lakes, NJ, USA).

### 4.7. Western Blot Analyses and Determination of CDK Activity

Total cell lysates were prepared and Western blot analysis was performed as described previously [44]. The relative intensity of each band was quantified using Image J software (NIH, Bethesda, MD, USA) and adjusted with its own β-actin. The control (0 μmol/L BITC) levels were set at 100%.

For *in vitro* CDK kinase activity assay, total cell lysates (0.75 mg protein) were immunoprecipitated using 1.5 μg of a polyclonal CDK2 or CDK4 antibody with protein-A-sepharose (Amersham) as described previously [44]. Immunoprecipitated proteins were incubated with a substrate (CDK2: Histone H1 (Roche, Basel, Switzerland) or CDK4: Rb (Santa Cruz Biotechnology)) and [γ-^32^P]ATP as previously described [43]. The resulting ^32^P-labeled histone H1 or RB was resolved on SDS-PAGE, and the gel was dried and subjected to autoradiography. The signals were quantitated via densitometric scanning of the film.

### 4.8. Statistical Analysis

The results are expressed as mean ± SEM. The results were analyzed using the general linear model (GLM), repeated measures analysis of variance (ANOVA) or one way ANOVA. Differences among the BITC treatment groups were statistically verified by conducting Duncan’s multiple-range test or Student’s *t*-test using the SAS system for Windows Version 9.2 (SAS Institute, Cary, NC, USA).

## Figures and Tables

**Table 1:** Effect of BITC on organ weights in mice.

BITC (mg/kg)	Normal Mouse		TRAMP Mouse		
	0	10	0	5	10
Liver weight	4.06 ± 0.05	4.1 ± 0.10	4.39 ± 0.17	3.99 ± 0.21	3.96 ± 0.22
Lung weight	0.58 ± 0.06	0.54 ± 0.02	0.59 ± 0.02	0.61 ± 0.02	0.61 ± 0.04
Spleen weight	0.27 ± 0.04	0.21 ± 0.02	0.27 ± 0.03	0.31 ± 0.03	0.29 ± 0.03

TRAMP mice and their nontransgenic littermates were exposed to BITC via gavage for 19 weeks as described in the materials and methods section. All mice were sacrificed at the age of 24 weeks and the livers, lungs, and spleens were excised from the mice and weighed. Values are expressed as the means ± SEM (normal mouse, *n* = 6; TRAMP mouse, *n* = 5).

**Table 2:** Effect of BITC on the levels of creatinine and activities of AST and ALT in the sera of mice.

BITC (mg/kg)	Normal Mouse		TRAMP Mouse		
	0	10	0	5	10
Creatinine (nmol/L)	0.20 ± 0.02	0.23 ± 0.05	0.25 ± 0.03	0.20 ± 0.01	0.18 ± 0.01
AST (U/L)	81.1 ± 6.47	59.9 ± 9.73	59.5 ± 3.69	52.8 ± 8.62	58.6 ± 8.89
ALT(U/L)	30.8 ± 14.0	9.50 ± 0.50	20.6 ± 5.13	21.3 ± 7.06	13.2 ± 1.74

Blood samples were collected from the mice, and the sera were prepared. The levels of creatinine and the activities of ALT and AST were measured using the appropriate assay kits. The values are expressed as the means ± SEM (normal mouse, *n* = 6; TRAMP mouse, *n* = 5).

## Abbreviations

ALT, alanine aminotransferase; AST, aspartate aminotransferase; BITC, benzyl isothiocyanate; CDK, cyclin-dependent kinase; DP, dorsolateral lobes of the prostate; GU tract; genitourinary tract; PIN, prostatic intraepithelial neoplasia; TRAMP, transgenic adenocarcinoma mouse prostate; WDC, well-differentiated carcinoma.
